# Combined treatment (image-guided thrombectomy and endovascular therapy with open femoral access) for acute lower limb ischemia: Clinical efficacy and outcomes

**DOI:** 10.1371/journal.pone.0225136

**Published:** 2019-11-15

**Authors:** Soo Buem Cho, Ho Cheol Choi, Sang Min Lee, Jae Boem Na, Mi Jung Park, Hwa Seon Shin, Jung Ho Won, Chung Eun Lee, Sung Eun Park

**Affiliations:** 1 Department of Radiology, Ewha Womans University Seoul Hospital, Ewha Womans University, College of Medicine, Seoul, Korea; 2 Department of Radiology, Gyeongsang National University School of Medicine and Gyeonsang National University Hospital, Jinju, Korea; 3 Thoracic and Cardiovascular Surgery, Gyeongsang National University School of Medicine and Gyeonsang National University Hospital, Jinju, Korea; 4 Department of Radiology, Gyeongsang National University School of Medicine and Gyeonsang National University Changwon Hospital, Changwon, Korea; University of Messina, ITALY

## Abstract

**Objectives:**

To evaluate the effectiveness and safety of combined treatment (image guided thrombectomy and endovascular therapy with open femoral access) for acute lower limb ischemia.

**Methods:**

From 2009 to 2017, 52 patients (44 men, eight women, mean 67.2 years) underwent combined treatment for acute thrombotic occlusion of lower extremity arteries. The patients presented with acute limb ischemia and we selectively perform combined treatment in the cases with challenging clinical considerations (e.g. various spectrum of thrombus, underlying atherosclerotic lesions). Combined treatment included cutdown of common femoral artery, thrombectomy using a Fogarty balloon catheter, balloon angioplasty, stenting, and catheter-introduced thrombus fragmentation and aspiration. Patients’ medical records were retrospectively reviewed and follow-up data were collected. The technical and clinical success rates and limb salvage were assessed. The Kaplan-Meier method was used to analyze primary patency rates and overall survival rates. Univariate analyses were performed to determine the factors related to clinical outcomes.

**Results:**

Technical and clinical success rate was 90.4% and 80.8%, respectively. The mean follow-up duration was 26.5 ± 25.8 months. Primary patency was 91.4%, 86.1%, and 74.6% at six months, 1-, and 2-year, respectively. Limb salvage without amputation was 88.5% (46/52). The overall survival rates at six months, 1-, and 3-year were 82.6%, 80.2, and 56.9%, respectively. The 30-day mortality was 5.8% (3/52). Univariate analysis showed that percutaneous transluminal angioplasty (PTA) type (balloon versus stent) was related to clinical failure.

**Conclusions:**

Combined treatment can be effective and safe for ALI patients even under challenging clinical conditions.

## Introduction

Acute limb ischemia (ALI) is the sudden decrease in limb perfusion due to either an embolic or a thrombotic vascular occlusion, which is defined as the presence of symptoms within two weeks of onset [1]. The profound ischemia represents an emergency in which delayed treatment results in limb loss and, potentially, death [2]. Therefore, timely recognition of an arterial occlusion and proper treatment for ALI are important. Traditionally, the treatment approach for ALI depends on the clinical categories based on TransAtlantic Inter-Society Consensus (TASC) [3]. Both surgical thrombectomy and endovascular treatment have benefits and drawbacks. Surgical thrombectomy using Fogarty embolectomy catheter has been the standard therapy because it is rapid and effective in cases of embolic thrombi in normal arteries [4]. However, blind surgical thrombectomy can result in poor revascularization or unexpected vascular injury in the presence of underlying arterial atherosclerosis or in the presence of subacute and chronic thrombi. In addition, thrombosis of runoff vessels are difficult to remove. Endovascular management (e.g. catheter-directed thrombolysis: CDT) has emerged as an alternative to surgery. It is less invasive, and does not directly damage the vascular endothelium with the capacity to clear thrombus in the small vessels. However, long treatment duration may worsen the clinical situation in advanced cases. Besides, a thrombus of more than two weeks does not respond well to the thrombolysis [5], and it is difficult to determine the exact stage of thrombus according to the clinical history. About 20% of patients have contraindications to thrombolytic therapy [6]. These problems may be minimized by combined treatment, which combines surgical thrombectomy and endovascular treatment. Unfortunately, a few previous studies were performed by vascular surgeons in which endovascular treatment was used as an adjunctive tool [7–9]. Furthermore, regarding combined treatment, there is little information as to its clinical efficacy or associated risk factors.

Here, we aimed to evaluate the effectiveness and safety of combined treatment for ALI with challenging clinical considerations (e.g. various spectrum of thrombus, underlying atherosclerotic lesions).

## Materials and methods

### Patient selection

The institutional review board of the University of Gyeongsang National University Hospital approved this retrospective study and waived the requirement for informed consent as we used only de-identified data collected as part of clinical practice (IRB No. 2019-10-012).

From June 2009 to May 2017, total 292 patients who presented at our institution with acute rising of the symptomatology suggestive for ALI were included in a dedicated database. Demographic and clinical data, as well as procedural details, were collected and analyzed retrospectively. Patients with acute ischemia due to graft occlusion (n = 73) and patients who underwent other management (e.g. surgery, conservative therapy) besides combined treatment (n = 167) were excluded from this study. The feasibility and benefits of combined treatment were discussed by a multidisciplinary team comprising of vascular surgeons and intervention radiologists. The inclusion criteria were as follows: 1) ALI (< 2 weeks). 2) Possibility of varied spectrum (e.g. acute-on-subacute) thrombosis when considering clinical symptoms and CT findings. 3) Presence of the underlying atherosclerotic lesions presumed to require PTA. 4) Thrombus in a poor location that is difficult to remove by surgical thrombectomy alone (e.g. thrombosis at runoff arteries). Finally, 52 consecutive patients who presented with acute lower limb ischemia and underwent combined treatment were enrolled.

### Procedure technique

**Fig 1:**
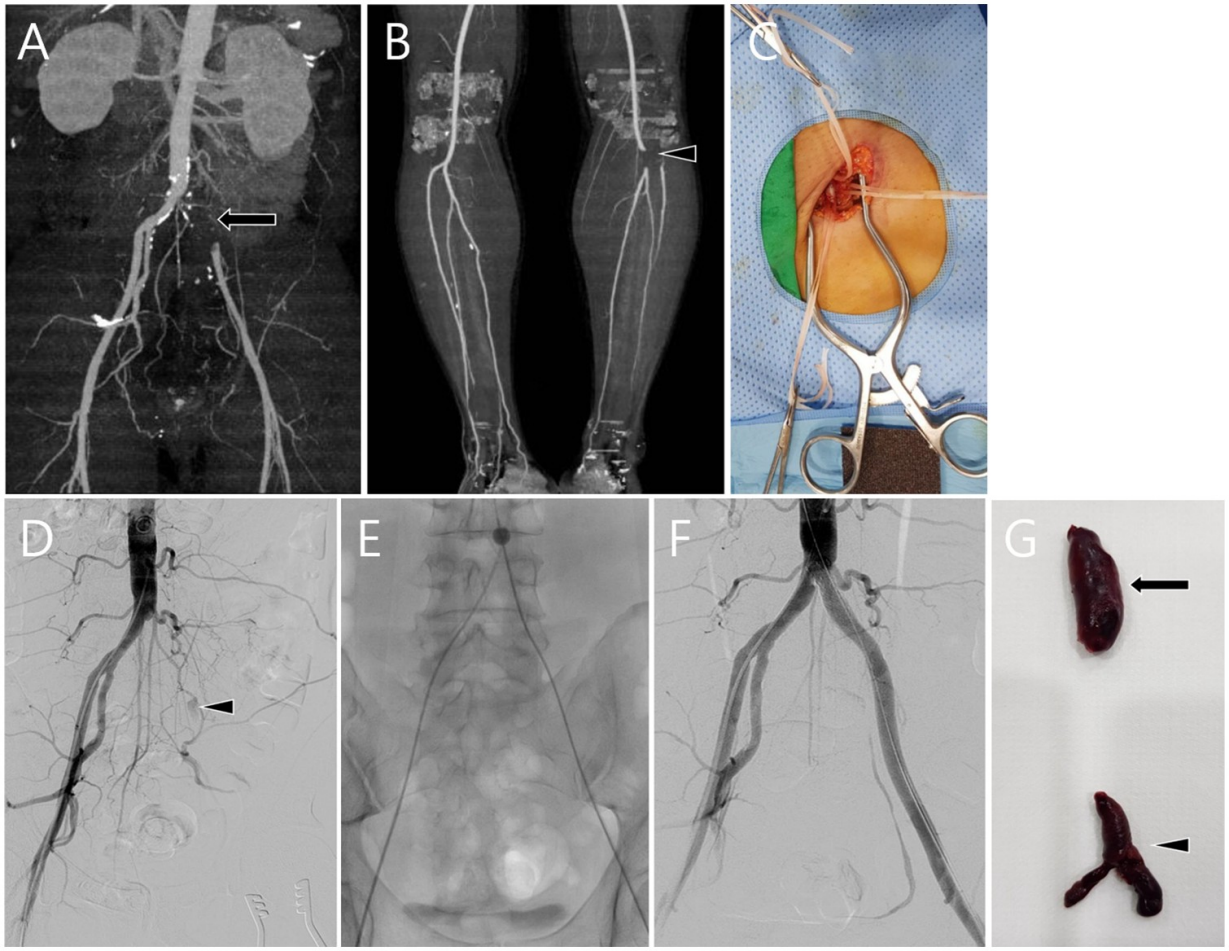
A 46-year-old man with acute limb ischemia presenting with cyanosis, coldness, and rest pain of left lower limb. There were no pulsation or Doppler signals on left femoral artery and dorsalis pedis artery. (A, B) Lower extremity angiography CT showed segmental occlusion of Lt common iliac artery (CIA) (arrow), proximal external iliac artery (EIA), proximal anterior tibial artery (ATA) (arrowhead), and tibioperoneal trunk. (C) Lt common femoral artery was cutdown state. (D) Aortoiliac arteriograms shows occluded left CIA and reconstituted EIA (arrowhead) from collateral flow. (E) Subsequent Fogarty thrombectomy was performed for Lt CIA and proximal runoff arteries occlusion. However, residual thrombosis and stenosis were noted at the Lt CIA, additional self-expanding stent was placed across the stenosis and ballooned (not shown). (F) Completion angiography demonstrated restoration of the flow at Lt CIA and proximal runoff arteries (not shown). (G) Gross appearance of the organized thrombus extracted from Lt CIA (arrow) and proximal runoff arteries (arrowhead).

When a diagnosis of ALI was confirmed by clinical examination, duplex ultrasound was performed by the on-call vascular surgeon. Subsequently, lower extremity angiography CT was performed as the basis for treatment decisions. If the vascular surgeons and interventional radiologists discussed a decision to combined treatment, it was performed as soon as possible. The procedure was performed under local anesthesia. All patients were approached through a common femoral artery (CFA) exposure (so-called cutdown) by a vascular surgeon in the angiography room, which permitted access for a balloon catheter thrombectomy from the aortic bifurcation to the proximal runoff arteries (Fig 1). Just before the arteriotomy, a bolus of 3000–5000 UI of heparin sodium was administered intravenously. After insertion of a 6-F introducer sheath via the exposed CFA, the thrombotic occlusion was gently crossed by a 4-F non-tapered Glidecath (Terumo, Somerset, NJ, USA). The Fogarty arterial embolectomy catheter (Edwards Lifesciences LLC, Irvine, USA) was used to facilitate the thrombectomy under fluoroscopic guidance. The appropriate size of the Fogarty balloon catheter was determined for the target artery (range, 2–5 Fr). We used sizes 4 and 5 Fr for proximal thrombectomy, and sizes 2 and 3 Fr for distal thrombectomy. The thrombectomy was continued until the appearance of strong back-bleeding from the recanalized artery and until no further thrombus could be removed via the Fogarty catheter. After closing the arteriotomy, the angiography was performed. When the angiography showed an incomplete restoration of perfusion, other endovascular options were considered. The catheter-introduced thrombus fragmentation, aspiration, and urokinase (UK) lysis were occasionally performed, which are finalized to clear the remaining intravascular thrombus. Aspiration thrombectomy was performed with a 5 Fr (KMP catheter; Cook Medical, Bloomington, IN, USA) to 6 Fr sized guiding catheter (Envoy Catheter; Codman & Shurtleff, Rhaynham, MA, USA), mainly for the crural artery. Catheter-directed thrombolysis was performed with an end hole or multiple side-hole thrombolytic infusion catheter (Multi-side port Catheter Infusion Set; Cook Medical, Bloomington, IN, USA). Urokinase was administered as initial bolus injection (100000–500000 units) and/or total amount of infused urokinase ranged from 500000 to 2000000 units. If the angiography revealed the presence of an underlying atherosclerotic steno-occlusive lesion, a plain balloon angioplasty was performed, and a metallic stent was implanted in case of residual stenosis >30% or flow-limiting dissections (bailout stenting). Primary stenting is also considered in cases of persistent partial intraluminal thrombosis adherent to the arterial wall after thrombectomy. CT angiography was routinely performed for all patients in this study, pre- and postoperatively.

### CT scanning

Several CT scanners were used during the 8-year study period as follows: the Brilliance 64, Ingenity 128 scanners (Philips Healthcare, Cleveland, OH, USA); Somatom Definition, the Somatom Definition flash scanners (Siemens Medical Systems, Erlangen, Germany); the LightSpeed VCT, Discovery 750 HD scanners (GE Healthcare, Milwaukee, WI, USA). The intravenous contrast medium (120–150 mL of 300–370 mgI/mL non-ionic contrast [Iopromide, Ultravist 300 or Ultravist 370; Bayer Healthcare, Berlin, Germany]) was administered at a rate of 3–5 mL/sec. The scanning delay for the arterial phase was 10–15 seconds after achieving enhancement of the descending aorta of up to 150 HU, as measured by an automatic bolus-tracking technique provided by the manufacturer of each CT scanner. The dynamic CT angiographic images were obtained during unenhanced, arterial, and venous phases. The scan parameters included beam collimation at 64 x 0.6 mm and 128 x 0.6 mm; beam pitch at 0.46–1.0; gantry rotation time of 0.5–0.8 seconds; field of view to fit; 120 kVp; automated dose reduction system (e.g. CARE Dose 4D; Siemens Medical Solutions, Erlangen, Germany) with the tube current set to 170–250 mAs. The images were reconstructed in the axial and coronal planes, ranging from a 1.25-mm thickness at 1.25-mm intervals to a 5-mm thickness at 5-mm intervals. Semiautomatic editing was performed for construction of maximum intensity projections using Aquarius 3D Workstation (TeraRecon Inc, San Mateo, Calif).

### Evaluation of data and definitions

Clinical outcomes, primary patency, and complications following revascularization were reported according to the “Recommended standards for reports” by Rutherford et al. [1]. ALI (less than 14 days) was defined as the sudden onset or worsening of ischemic manifestations within the lower extremities due to arterial thrombosis or embolism. Category I refers to viable limbs. Category IIa is marginally threatened limb and, if properly treated, salvageable. Category IIb is immediately threatened limb that require immediate revascularization if salvage is to be accomplished. Category III is irreversibly damaged limb. In this, major tissue loss or permanent nerve damage is inevitable. Technical success was defined as continuous arterial patency of at least one artery to the foot without any obvious flow-limiting lesions (absence of a stenosis >30%). Clinical success was defined as no recurrence of acute limb ischemia, restenosis or reocclusion of a treated arterial segment within one month after combined treatment. Limb salvage was defined as freedom from major amputation above the foot level. Loss of patency was determined by follow-up clinical symptoms, CT angiography, and/or Doppler ultrasound findings of occlusion in the treated arterial segment. The overall survival period was defined as the interval, in months, between the initial combined treatment and the patient’s death. Complications were categorized as major or minor, according to the guidelines of the Society of Interventional Radiology (SIR) Standards of Practice Committee [10]. Major complications were defined as complications requiring major therapy, complications necessitating an unplanned increase in the level of care or prolonged hospitalization (>48h), and complications resulting in permanently adverse sequelae or patient death. Minor complications were defined as complications requiring no therapy or only nominal therapy, including overnight hospital admission for observation. Acute thrombosis refers to symptoms that have been present for less than 14 days or imaging studies indicating that thrombus occurred within the previous 14 days. Subacute thrombosis refers to symptoms or thrombus that have been presented for 15–28 days as indicated by history or imaging studies. Chronic thrombosis refers to symptoms or thrombus having been presented for more than 28 days, as indicated by history or imaging studies [11].

### Statistical analysis

The Kaplan-Meier method was used to analyze primary patency rates and overall survival rates. Outcomes were compared between successful and failed treatment. To determine the factors associated with clinical success, univariate analyses were performed. Univariate analysis of the categorical data was assessed using the Fisher exact test or χ^2^ test, and univariate analysis of continuous data was assessed using the Mann-Whitney test. P values less than .05 were considered to indicate statistical significance. All statistical analyses were performed using the Statistical Package for the Social Sciences (SPSS) for Windows, version 21.0 (SPSS, Inc., Chicago, IL, USA).

## Results

### Patient characteristics

The patient and limb-baseline characteristics of the 52 patients (44 men, eight women; mean age: 67.2 ± 14.3 years) undergoing combined treatment due to ALI are summarized in Table 1. Of the 52 patients, seven (13%) had arterial fibrillation or arrhythmias, four (8%) had a history of ischemic cardiopathy (e.g. acute myocardial infarction), eight (15%) had a history of cerebrovascular disease (e.g. acute cerebral infarction), four (8%) had a previous revascularization of a lower limb (e.g. SFA stent due to chronic total occlusion, surgical thrombectomy due to popliteal artery thromboembolism, PTA due chronic limb ischemia, and Kissing-stenting due to Leriche syndrome). Duration of symptoms was more than 24 hours for 44 patients, between six and 24 hours for six patients, and within six hours for two patients. As to the clinical category of ALI, 13 patients were stage I, 18 patients were stage IIa, 19 patients were stage IIb, and two patients were stage III.

**Table 1:** Patient and limb baseline characteristics.

Characteristic	Value (n = 52)	Success (n = 42)	Failure (n = 10)	P value
Age (y) (range)	67.2 (37–93)	68.2 (37–93)	63.2 (42–83)	0.429
Gender, male/female	44/8	35/7	9/1	1.000
Current/former smokers	31 (60)	26 (62)	5 (50)	0.500
Diabetes	12 (23)	9 (21)	3 (30)	0.679
Hypertension	23 (44)	21 (50)	2 (20)	0.155
Atrial fibrillation/arrhythmias	7 (13)	5 (12)	2 (20)	0.607
Cerebrovascular disease	8 (15)	7	1	1.000
Ischemic cardiopathy	4 (8)	3	1	1.000
Previous revascularization lower limb(s)	4 (8)	4	0	0.576
Duration of symptoms				0.999
*0–6 hours*	2	2	0	
*6–24 hours*	6	5	1	
*>24 hours*	44	35	9	
Clinical category of ALI				0.065
*Stage I*	13	13 (31)	0	
*Stage IIa*	18	15 (36)	3 (30), *1	
*Stage IIb*	19	13 (31)	6 (60), * 4	
*Stage III*	2	1 (2)	1 (10), *1	

*ALI* acute limb ischemia,

* indicates number of the patient underwent amputation.

### Outcome of combined treatment

The procedural details were summarized in Table 2. Thrombosis locations were classified as follows: iliac (I), two patients; femoropopliteal (FP), 17 patients; tibial (T), one patient; multilevel, 32 patients. PTA locations were classified as follows: none, three patients; I, seven patients; FP, six patients; T, 15 patients; multilevel, 21 patients. PTA types were classified as follows: none, three patients; balloon, 22 patients; stenting, 27 patients. Aspiration thrombectomy, catheter-directed thrombolysis, and both were performed in 11 patients, three patients, and four patients, respectively.

**Table 2:** Procedural details of combined treatment.

Characteristic	Value (n = 52)	Success (n = 42)	Failure (n = 10)	P value
Thrombosis location				0.254
*Iliac*	2	2	0	
*Femoropopliteal*	17	15	2	
*Tibial*	1	0	1	
*Multilevel*	32	25 (I-FP, 8; I-T 1; FP-T, 14; I-FP-T, 2)	7 (all FP-T)	
PTA location				0.205
*None*	3	3	0	
*Iliac*	7	7	0	
*Femoropopliteal*	6	6	0	
*Tibial*	15	10	5	
*Multilevel*	21	16 (I-FP, 6; I-T 1; FP-T, 9)	5 (all FP-T)	
PTA type				0.036
*None*	3	3	0	
*Balloon*	22	14	8	
*Stenting*	27	25	2	

*I* iliac artery, *FP* femoropopliteal, *T* tibial, *PTA* percutaneous transluminal angioplasty.

**Fig 2:**
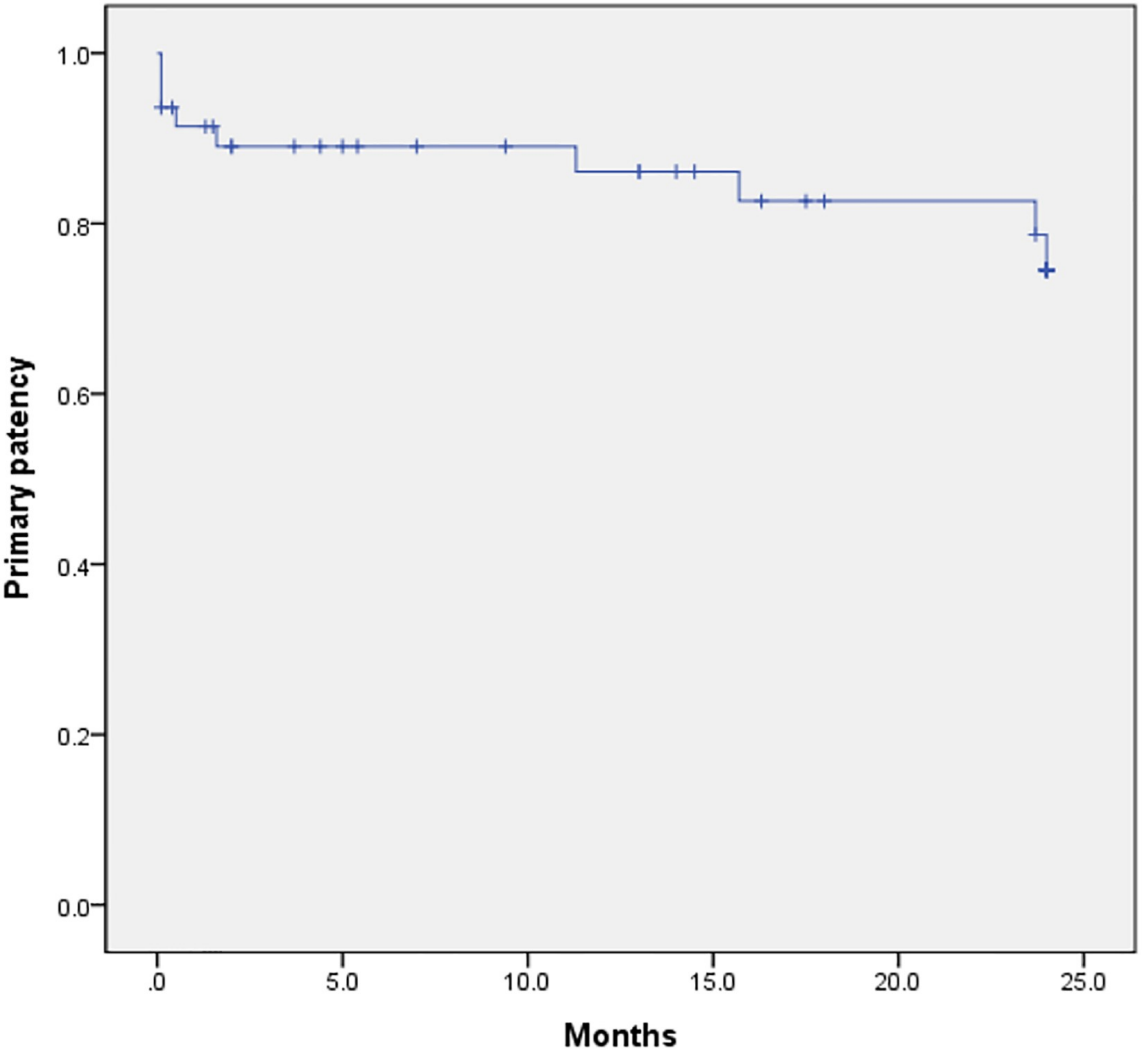
Graph shows primary patency of the lesions treated by combined treatment.

**Fig 3:**
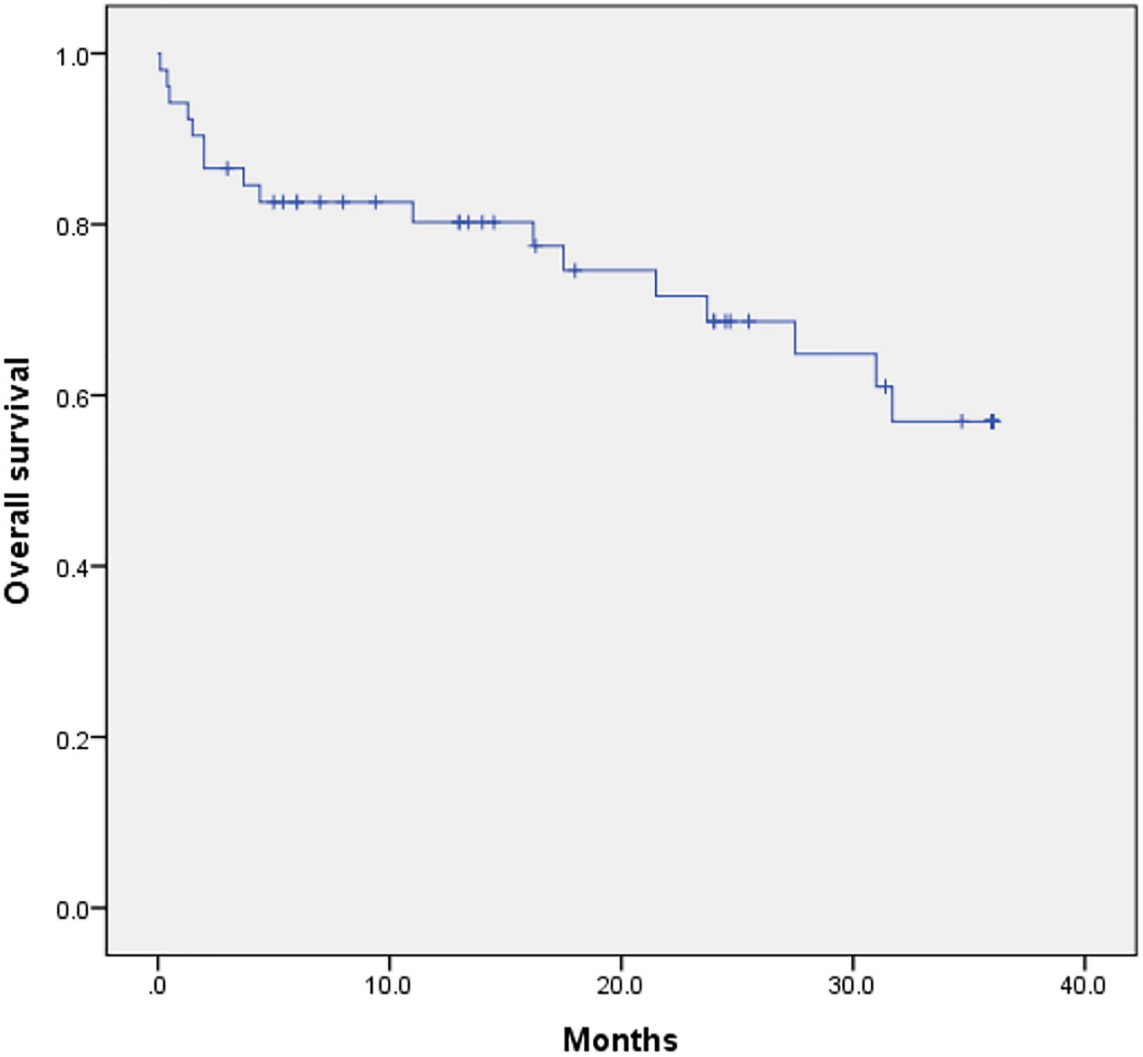
Graph shows cumulative overall survival of the patients treated by combined treatment.

Technical and clinical success rates were 90.4% (47/52) and 80.8% (42/52), respectively. Of 10 clinical failures, one patient underwent reintervention (balloon angioplasty with stenting), one patient did not undergo additional procedure because of the expectation of a collateral supply, two patients were rejected for amputation and discharged (one died two weeks later, and the other one was lost from follow-up), six patients underwent major amputations. All amputations occurred in those patients with clinical failure. The mean follow-up duration was 26.5 ± 25.8 months (range: 0.1–99 months). Primary patency was 91.4%, 86.1%, and 74.6% at six months, 1-year, and 2-year, respectively (Fig 2). Limb salvage without amputation was 88.5% (46/52). The overall survival rates at six months, 1-year, and 3-year were 82.6%, 80.2%, and 56.9%, respectively (Fig 3). The incidence of death within 30 days after combined treatment (30-day mortality) was 5.8% (three of 52). Causes of death were as follows: multiple organ failure two weeks after technical failure and patient’s rejection of amputation; acute kidney injury due to rhabdomyolysis, suggestive of reperfusion injury; comorbidity such as acute exacerbation of chronic obstructive pulmonary disease.

Univariate analysis showed that PTA type (balloon alone versus stenting) was related to clinical failure (P = 0.036). Among the 52 patients, 39 out of 42 clinical success and all 10 clinical failure had underlying stenotic lesions that required PTA. In the 27 patients (clinical success, 25 patients: clinical failure, 2 patients), the stents were implanted at iliofemoral arteries except tibial arteries, whereas the 22 patients (clinical success, 14 patients: clinical failure, 8 patients) underwent only balloon angioplasty. In the three other patients, the unmasked focal lesion was managed conservatively. Clinical success was not associated with age, gender, smoking, diabetes, hypertension, arterial fibrillation/arrhythmias, cerebrovascular disease, ischemic cardiopathy, previous revascularization of lower limbs, duration of symptoms, clinical category of ALI, thrombosis location, or PTA location.

There were complications in 12 patients (23%): three hematomas at the cutdown site, five arterial dissection, and five extravasations (one patient had both arterial dissection and extravasation). In five patients, superficial femoral artery dissection limited flow, and this required stent placement after failed long-duration balloon inflation. In five patients, arterial extravasation (one popliteal a., three peroneal a., one posterior tibial a.) were due to Fogarty balloon thrombectomy. Popliteal artery was treated with embolization and stenting, one peroneal artery was embolized with microcoils. Two peroneal arteries and one posterior tibial artery could be treated with long-duration balloon inflations.

## Discussion

Acute occlusion of peripheral arteries pose a significant risk to limb and life. The fluoroscopically assisted thromboembolectomy has been known to improve the technical success of hybrid treatment for ALI [7–9]. Unfortunately, the role of combined treatment for patients presenting with ALI has not yet been established. The management of ALI usually depends on the clinical stage in the TASC document [3]. In stage I (viable) and IIa (marginal threatened), thrombolysis as well as surgical thrombectomy are accepted treatment options. In stage IIb (immediate threatened), surgical thrombectomy is the treatment of choice. In early stage III (irreversible), surgical thrombectomy may be applied, but in advanced stage III, amputation is the treatment of choice. Our primary targets of ALI patients are an acute-on-subacute thrombosis (which is difficult to remove with aspiration thrombectomy or catheter-directed thrombolysis) and/or stage IIa, IIb, early III cases with pre-existing arterial lesion (which requires rapid revascularization, but if there is an underlying disease, revascularization is incomplete). Our experience showed the central role of combined treatment in the management of ALI with ambiguous age of thrombus and/or underlying artherosclerosis.

In the current study, the technical and clinical success rates were 90.4% (47/52) and 80.8% (42/52), respectively. Primary patency at 1-year was 86.1%. This study showed that combined treatment for ALI with acute and/or subacute thrombosis can be effective. In the previous randomized controlled trials dealing with outcomes of ALI treatment (operative or CDT), revascularization successes were 70% to 79.7% [6,12,13]. Our high technical success is attributed to advanced endovascular techniques and supplementing for each other’s shortcomings in combined treatment. In the prior literatures, intraoperative angiography after surgical thrombectomy demonstrated 25–40% residual thrombus, presence of underlying steno-occlusive lesions, or incomplete restoration of runoff arteries [14,15], which may be responsible for the early failure. Our study confirmed that detection of imperfect revascularization needing additional treatment was very high (94%, 49/52) because thrombus over subacute age was occasionally not removed, even though using Fogarty balloon catheter, and there were frequent underlying steno-occlusive lesions. In particular, PTA of runoff arteries was associated with clinical failure. The incidence of runoffs PTA in the clinical success versus clinical failure were 60% (25/42) and 100% (10/10), respectively. Distal runoffs are known to be important factors for the patency of SFA lesion, which was treated by PTA [16]. Thus, revascularization of ALI may be ineffective in the case of absent distal arteries, even after primary in situ thrombectomy.

Our limb salvage rate (88.5%, 46/52) was comparable, and 30-day mortality (5.8%, 3/52) was lower than that of operative treatment, which, in the preliminary literatures, has ranged from a 70%–84% limb salvage rate with 13%–22% mortality [17–20]. Traditionally, the decisional breakpoint in choosing between endovascular and surgical revascularization came between stages IIa and IIb. The combination of surgical and endovascular options may overcome the limitations of the traditional approach, which can be used in the Rutherford clinical category IIb. Therefore, combined treatment, as rapid revascularization with use of the less invasive technique, can be the cornerstone of successful therapy to minimize the amputation rate and mortality.

Univariate analysis showed PTA type was related to clinical failure in the current study. Of the ten patients with clinical failure, eight underwent balloon angioplasty alone, while two underwent iliofemoral arterial stenting. It might be associated that all clinical failure cases had tibial arterial lesions which was difficult to stent implantation. However, stenting appears to achieve better technical success, complication control, and short-term primary patency in the ilio-femoro-popliteal lesions than balloon angioplasty alone [21–24]. Recent report demonstrates such a drug-eluting stent reduces the risk of reintervention and amputation even in infrapopliteal arteries [25]. Therefore, it may be useful to give proper consideration to stent placement in the situation of suboptimal balloon PTA results for the steno-occlusive lesions of ALI. Even though clinical category of ALI was not showed statistical significancy, all stage I had clinical success, and there is tendency of high clinical failure in the advanced clinical category. Thus, patient’s selection for combined treatment is also important for beneficial outcomes.

Our experience showed that endovascular treatment after thrombectomy can be effective in the incomplete recanalization of occluded arteries due to residual thrombus with strong adhesion to the arterial wall, residual thrombus in distal vessels, or presence of underling steno-occlusive lesions after clot removal. Additionally, most complications related to combined treatment were minor, and all could be treated at the same endovascular session. Consequently, we believe that combined treatment is the effective and rapid solution for removing a large and organizing clot in the acute ischemic limbs, even with underlying steno-occlusive lesions.

Our study has several limitations. First, it was of retrospective design based on a review of medical records. Second, the statistical power for the comparison between clinical success and clinical fail is low because the sample size of the latter group was small (n = 10). Third, combined treatment was performed selectively based on subjective criteria. This may have introduced bias in favor of combined treatment. Fourth, the age of thrombus was not pathologically confirmed but was estimated by the symptoms and CT findings.
